# Mean Platelet Volume in the Diagnosis of Acute Appendicitis in the Pediatric Population: A Systematic Review and Meta-Analysis

**DOI:** 10.3390/diagnostics12071596

**Published:** 2022-06-30

**Authors:** Nellai Krishnan, Sachit Anand, Niklas Pakkasjärvi, Minu Bajpai, Anjan Kumar Dhua, Devendra Kumar Yadav

**Affiliations:** 1 Department of Pediatric Surgery, All India Institute of Medical Sciences, New Delhi 110029, India; nellai93@gmail.com (N.K.); bajpai2b@gmail.com (M.B.); anjandhua@hotmail.com (A.K.D.); drdevendra@hotmail.com (D.K.Y.); 2 Department of Pediatric Surgery, Kokilaben Dhirubhai Ambani Hospital, Mumbai 400053, India; drsachit_anand@outlook.com; 3 Department of Pediatric Surgery, Turku University Hospital and University of Turku, 20521 Turku, Finland; 4 Department of Pediatric Surgery, Uppsala Akademiska Sjukhuset, 751 85 Uppsala, Sweden

**Keywords:** acute appendicitis, non-specific abdominal pain, children, mean platelet volume, biomarker

## Abstract

Background: Mean Platelet Volume (MPV) has been suggested as a biomarker for acute appendicitis (AA) in the adult population. The utility of MPV in pediatric AA remains vague. This systematic review and meta-analysis aimed to systematically summarize and compare all relevant data on MPV as a diagnostic biomarker for AA in children. Methods: Databases were systematically searched using keywords ((mean platelet volume) OR mpv) AND (appendicitis). The inclusion criteria were all comparative studies of MPV in children aged less than 18 years and diagnosed with AA. Two authors independently assessed the methodological quality using the Downs and Black scale. Results: We included fourteen studies in the final meta-analysis; most were retrospective. Eight studies compared the MPV values between AA and non-AA; four studies compared the same between AA and healthy controls; two studies compared the MPV values among all three groups. The estimated heterogeneity among the studies for all outcomes was high and statistically significant. The pooling the data showed no statistically significant difference in MPV (weighted mean difference (WMD) = −0.42, 95% CI = (−1.04, 0.20), *p* = 0.19) between AA and healthy controls or AA and non-AA cases (WMD = 0.01, 95% CI = (−0.15, 0.17), *p* = 0.90). Conclusion: While MPV levels seem to have some utility in adult AA diagnosis, MPV levels should not dictate treatment options in pediatric AA.

## 1. Introduction

Acute appendicitis (AA) remains one of the most common indications for emergency room evaluations among children who present with abdominal pain [1]. Despite advances in investigative modalities, AA still presents diagnostic challenges. Delays in diagnosis and subsequent management increase the risk of morbidity among affected patients with complications including appendix rupture, abscess, peritonitis, and sepsis [2]. Acute appendicitis initiates a humoral immune response, which can be detected through several biomarkers [3]. Traditionally, clinical status complemented by elevated leukocyte and C-reactive protein levels has been used to diagnose AA. Still, the sensitivity and specificity of these markers remain insufficient by themselves [4]. Radiological modalities are commonly employed, with some centers using Magnetic Resonance Imaging (MRI) for definitive diagnosis. Each investigation raises costs and adds further delays to diagnosis, thereby postponing possible intervention. Various scoring systems have also been developed to refine diagnostic accuracy. Recently, in addition to white blood cell count (WBC), blood parameters have shown promise in increasing specificity for diagnosing AA [5]. A recent systematic review and meta-analysis on the utility of Red Cell Distribution Width (RDW) in AA showed no evidence of RDW values in diagnostic testing of AA [6]. Mean platelet volume (MPV) is related to thrombocyte function and activation and varies in response to humoral immune reactions. The reactions of platelet indices to inflammatory conditions have been investigated in depth, and especially the role of MPV as an acute phase reactant and its relation to platelet count (PC) has raised interest in the diagnosis of AA [7,8].

Despite many reports on the utility of complete blood cell count in AA diagnostics, thus far, the utility of MPV in children remains vague. We hypothesized that the MPV levels would provide additional substance for a better diagnosis of AA in children. To that end, we conducted a systematic review on MPV in diagnosing AA in the pediatric population. To our best knowledge, this is the first systematic review and meta-analysis on the utility of MPV in AA diagnostics among children.

## 2. Materials and Methods

### 2.1. Search Strategy

The literature search was conducted as per the Preferred Reporting Items for Systematic Reviews and Meta-Analyses (PRISMA) guidelines [9]. Two investigators (NK and SA) independently conducted searches on PubMed, Web of Science, EMBASE, and Scopus databases on 20 March 2022. The search keywords used were ((mean platelet volume) OR mpv) AND (appendicitis). The total search records were analyzed, and duplications were removed. Subsequently, the eligibility criteria were applied to screen the relevant studies.

### 2.2. Eligibility Criteria

The inclusion criteria were: all patients aged less than 18 years with pathological confirmed acute appendicitis (AA). The non-AA patients included those patients with either negative appendectomy by pathology or non-specific abdominal pain (NSAP). Healthy controls were asymptomatic individuals. The MPV was compared between AA, non-AA patients, and healthy controls. Studies with incomplete data, or where the outcomes of interest were not reported, were excluded. Additionally, those comparing only the severity or stage of appendicitis with MPV were excluded. Case reports, literature reviews, commentaries, editorials, conference abstracts, and opinion articles were also excluded.

### 2.3. Data Extraction

Two investigators (NK and SA) independently performed data synthesis using Microsoft Excel spreadsheets. Extracted information included the first author’s name, year of publication, sample size, age, gender, and mean ± standard deviation (SD) of MPV in cases and controls. Disagreements were settled by discussion and consensus with the senior authors (NP, MB).

### 2.4. Quality Assessment

An independent assessment of the methodological quality was performed by two authors (AD and DKY) using the Downs and Black scale [10]. This validated 27-point scale has four domains of assessment with minimum and maximum scores of 0 and 32, respectively. Based on these scores, the risk of bias was graded as low (score > 23), moderate (16–23), or high (0–15). The kappa statistics were used to identify the level of inter-rater agreement regarding the risk of bias [11]. The degree of agreement could be graded as slight (0.00–0.20), fair (0.21–0.40), moderate (0.41–0.60), substantial (0.61–0.80), and almost perfect (0.81–1.00).

### 2.5. Statistical Analysis

During this study, the guidelines from the Cochrane handbook were followed [12]. RevMan 5.4 (Cochrane Collaboration, London, UK) software was used to perform the meta-analysis. All numerical data are shown as mean ± SD. As both the study outcomes were continuous, mean differences (MD) were calculated for them. Subsequently, the inverse variance (IV) method was used to calculate the weighted mean difference (WMD). The I^2^ statistics were applied for the analysis of heterogeneity. Significant heterogeneity was interpreted when I^2^ was >50%. A random-effects model was used in case of substantial heterogeneity (I^2^ > 50%). A value of *p* < 0.05 was considered statistically significant.

### 2.6. Research Ethics and Consent

Due to the nature of this study, where data was collected from previous clinical studies and synthesized subsequently, ethical aspects remain methodological, and ethical approval, patient confidentiality and informed consent were unapplicable for this study.

## 3. Results

### 3.1. Study Characteristics

**Figure 1:**
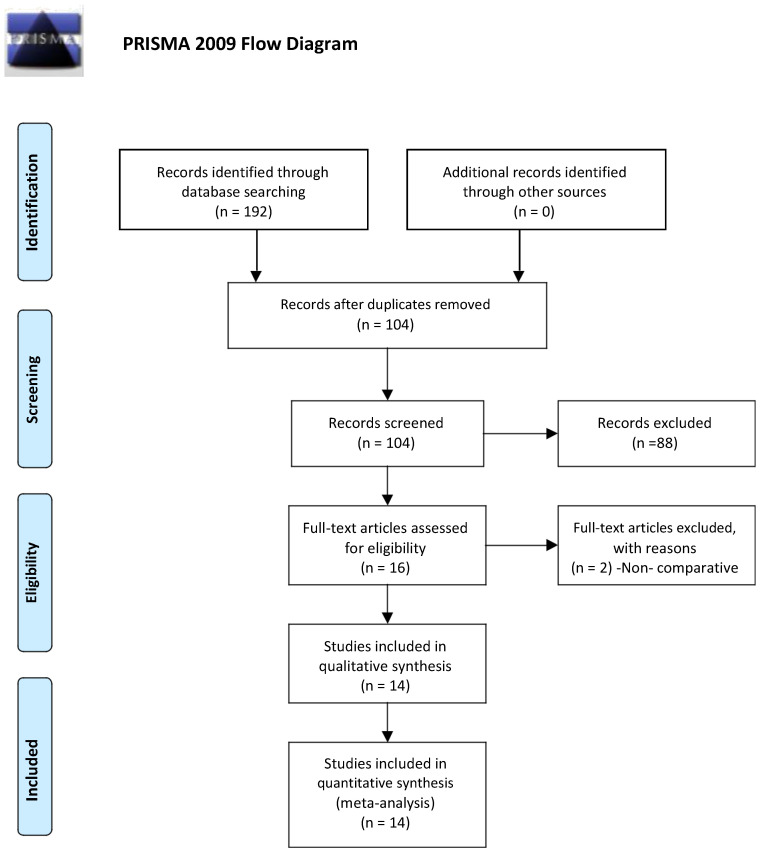
Selection of the relevant studies using the Preferred Reporting Items for Systematic Review and Meta-Analysis (PRISMA) flow diagram.

Our search strategy identified 192 records. After the removal of 88 duplicates, 104 articles were screened for eligibility. Of these, 88 abstracts were excluded, and 16 full-text articles were assessed using the inclusion criteria (Figure 1). Two studies were non-comparative, so they were further excluded. Finally, 14 studies were included in the meta-analysis. The study designs of these studies were retrospective (*n* = 10), cross-sectional (*n* = 1), case-control (*n* = 1), and prospective study (*n* = 2). Eight studies compared the MPV values between AA and non-AA only; four studies compared the same between AA and healthy controls, while two studies compared the MPV values among all three groups. A total of 3796 subjects comprising 2461 with AA, 557 with NSAP, and 778 healthy controls were included in the meta-analysis. The baseline characteristics of the included studies are demonstrated in Table 1.

### 3.2. Summary of the Included Studies

- **Bilici et al., 2011**

This retrospective study was conducted in Turkey [13]. The sample size was 100 patients with AA and 100 healthy controls. The study found that MPV values significantly decreased (*p* < 0.001) in children with AA. The study also investigated the role of white blood cell (WBC), neutrophil and platelet counts.

- **Uyanik et al., 2012**

This retrospective study was conducted in Turkey [7]. It included 305 patients with AA and 305 healthy controls. The study found no statistically significant difference in MPV values (*p* > 0.05) between the two groups. The study also investigated the leukocyte count between the two groups.

- **Nazik et al., 2017**

This prospective study was also conducted in Turkey [14]. The sample size included 63 patients (30 AA and 30 healthy controls). The study found that MPV values did not significantly differ between the two groups (*p* = 0.501). The study also investigated the role of WBC, C-reactive protein (CRP), the neutrophil lymphocyte ratio, the platelet lymphocyte ratio, and erythrocyte sedimentation rate (ESR).

- **Yilmaz et** **al., 2017**

This retrospective study from Turkey on 628 patients with AA and 30 in non-AA groups showed that the MPV values did not significantly differ between the two groups (*p* = 0.498) [15]. The study also investigated the role of WBC, neutrophil count, lymphocyte count, hemoglobin, hematocrit, platelet count, red cell distribution width (RDW), and CRP.

- **Yazar et al., 2018**

This cross-sectional study from Turkey was primarily aimed at assessing the reliability of ultrasonography and the Alvarado scoring system in acute appendicitis [16]. The sample size included a total of 200 patients (170 AA and 30 non-AA). The study also showed that MPV values did not significantly differ between the two groups (*p* = 0.830). The study also reported the values of WBC, platelet count, CRP, and absolute neutrophil count (ANC) in the two groups.

- **Nia et al.,** **2018**

This case-control study was conducted in Ian [17]. The sample size was 60 cases of AA and 60 controls. The study found that there was no significant difference in MPV values between the two groups (*p* > 0.05). The study also investigated the role of other biomarkers such as ESR, WBC, lymphocyte count, and the polymorphonuclear leukocytes (PMNs) percentage.

- **Bozlu et al.,** **2019**

This retrospective study was conducted in Turkey [18]. The sample size was 219 children (141 with AA, 46 non-AA, 32 perforated AA), and 100 healthy children. The study found no significant difference in the MPV values between the groups (*p* = 0.663). The other markers investigated in the study were WBC, platelet count, platelet distribution width (PDW), mean platelet volume-to-lymphocyte ratio (MPVLR), platelet-to-lymphocyte ratio (PLR), and CRP.

- **Tuncer et al.,** **2019**

This retrospective study from Turkey on 137 patients with AA found that MPV values differed significantly among the study groups, which also included children with familial Mediterranean fever and mesenteric lymphadenitis [19]. The study also investigated the role of the neutrophil/lymphocyte ratio, the platelet/lymphocyte ratio, and the lymphocyte/monocyte ratio.

- **Oktay et al.,** **2020**

This retrospective study was conducted in Turkey. It included 207 pediatric patients who were divided into three groups (non-AA, uncomplicated AA, and complicated AA) [20]. The mean MPV was highest in the non-AA group, and there was a significant difference in the MPV among the groups (*p* = 0.047). The study also analyzed the role of WBC and the MPV/platelet count (PC) ratio.

- **Sengul et al.,** **2020**

This cross-sectional study from Turkey on 205 patients with AA and 30 negative appendectomy patients found that MPV values did not significantly differ between the groups [21]. The study also investigated the role of WBC, neutrophil count, lymphocyte count (LC), the neutrophil/lymphocyte ratio (NLR), RDW, and CRP.

- **Du et al., 2020**

This prospective study from China was primarily aimed at studying the role of soluble B7H3 (sB7H3) in AA and its accuracy as a predictor of the severity of appendicitis [22]. The sample size included 92 patients with AA and 90 healthy controls. The study also showed that the MPV values were significantly lower in AA as compared to healthy controls (*p* < 0.001). The study also reported the values of other laboratory markers such as WBC, RDW, CRP, platelet count, and platelet distribution width (PDW).

- **Tartar et al., 2020**

This retrospective study from Turkey on 186 children with confirmed AA showed no significant difference in the MPV values (*p* = 0.536) between AA and non-AA groups [23]. The study also evaluated the role of other laboratory results like WBC, NLR, RDW, CRP, and procalcitonin levels.

- **Duman et al., 2022**

This retrospective study was conducted in Turkey. The sample size included 254 patients with AA, 197 with non-AA, and 150 controls [24]. The study found no significant difference in the mean platelet volume (MPV) between the groups. Other markers investigated were the monocyte-to-lymphocyte ratio, the platelet-to lymphocyte ratio (PLR), the neutrophil-to-lymphocyte ratio (NLR), WBC, neutrophil percentage, CRP, and sodium level.

- **Dooki at al., 2022**

This retrospective study was conducted in Iran [25]. A total of 100 patients (50 with AA and 50 with non-AA) were studied. The study showed that the mean value of MPV in children with non-AA was higher than in children with AA, and this difference was significant (*p* < 0.001). Other markers investigated in this study include WBC, CRP, ESR, and PMNs.

### 3.3. Methodological Quality Assessment

The Downs and Black scoring by two authors are depicted in Table 2. All studies had a low risk of bias. The average Downs and Black scores for the included studies ranged from 24–28. The studies by Tartar et al. and Tuncer et al. had the minimum and maximum scores, respectively [19,23]. The inter-observer agreement was almost perfect (Kappa = 0.928, *p* < 0.0001).

### 3.4. Outcome Analysis

#### 3.4.1. MPV Values in Children with AA Versus Healthy Controls

**Figure 2:**
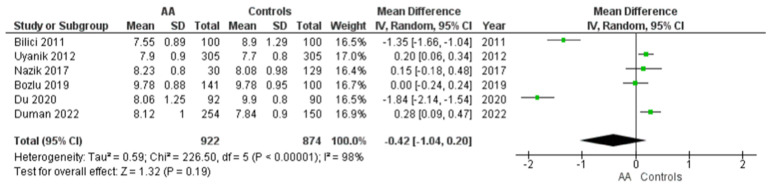
Forest plot comparison between the two patient groups (AA versus healthy controls) in terms of the average values of mean platelet volume. Abbreviations: AA, acute appendicitis; IV, inverse variance; CI, confidence interval.

Six studies reported this outcome. The MPV values of a total of 922 patients with acute appendicitis and 874 healthy controls were compared. The estimated heterogeneity amongst the studies for this outcome was statistically significant (I2 = 98%, *p* < 0.00001). Pooling the data (Figure 2) using random-effects model demonstrated no statistically significant difference in the MPV values (Mean difference = −0.42, 95% CI = [−1.04, 0.20], *p* = 0.19) between children with AA and healthy controls.

#### 3.4.2. MPV Values in Children with AA Versus Non-AA

**Figure 3:**
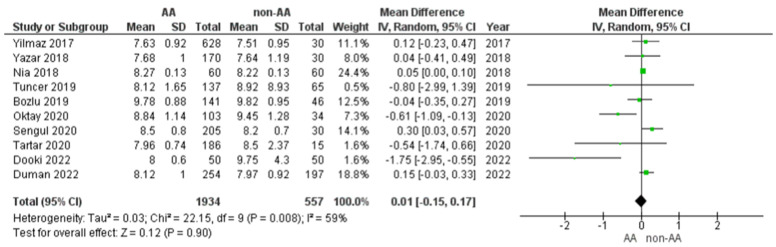
Forest plot comparison between the two patient groups (AA versus non-AA) in terms of the average values of mean platelet volume. Abbreviations: AA, acute appendicitis; non-AA, non-acute appendicitis; IV, inverse variance; CI, confidence interval.

Ten studies reported this comparison. The MPV values of a total of 1934 patients with acute appendicitis and 983 NSAP were compared. The estimated heterogeneity amongst the studies for this outcome was statistically significant (I2 = 59%, *p* = 0.008). Pooling the data (Figure 3) using random-effects model demonstrated no statistically significant difference in the MPV values (Mean difference = 0.01, 95% CI = [−0.15, 0.17], *p* = 0.90) between AA and non-AA.

## 4. Discussion

Acute appendicitis presents challenges, and alternative methods for diagnostic verification are ongoing. While severe appendicitis is treated by appendectomy, the treatment choices for the mild disease remain pendent, and some cases may resolve without any intervention [26]. It has been suggested that complicated and uncomplicated appendicitis are at the very ends of the disease spectrum and may even be considered different diseases [27]. Especially in children, a significant proportion of patients presenting with lower abdominal pain do not have AA. Nevertheless, those who have may progress to complicated appendicitis rapidly, underlining the importance of correct diagnosis for appropriate treatment. Thus, the diagnosis must be verified by methods with good sensitivity and high negative predictive value without accompanying delay for accurate treatment initiation.

While radiological methods are improving, they are not without challenges. Computed tomography has been validated in differentiating between complicated and uncomplicated AA but entails ionizing radiation, which is to be avoided, especially in children [28]. Ultrasonography avoids the radiation exposure of CT but is not sufficient as the sole diagnostic method in the differentiation of complicated versus uncomplicated AA [29]. The use of MRI presents with high sensitivity and specificity but entails costs and is not universally available in centers that treat children with AA [30].

Several biomarkers can detect the humoral immune response to AA, but none of them are qualitative enough to be utilized individually. To enhance the diagnostic accuracy of AA without subsequent economic burden, we wanted to focus on the role of traditional biomarkers. Whole blood cell count, especially erythrocyte-related measurements, has raised interest and shown some promise in diagnosing AA. Inflammatory states influence MPV levels, with increases usually found in chronic diseases and decreases seen in acute conditions [31]. The MPV is influenced by cytokines such as interleukin (IL)-1 and IL-6, but variations in platelet indices are more general markers of inflammation than specific markers for AA. In this systematic review and meta-analysis, we aimed to evaluate the utility of mean platelet volume for diagnostic purposes in AA. The results demonstrated that MPV values did not present significant changes in either distinguishing AA from healthy children or AA from non-specific abdominal pain. Fan et al. performed a systematic review and meta-analysis on the correlation between AA and MPV and concluded that MPV was lower in patients with AA [8]. Tullavardhana et al. looked at platelet indices in AA in a meta-analysis and suggested that lower MVP values could function as a marker of AA [32]. Shen et al. concluded in their meta-analysis that only MPV, but no other platelet indices, vary in AA cases [33]. In the subgroup analysis of the same study, MPV levels seem to decrease in AA patients over 30 years of age, but this cohort was limited in number [34]. These studies included patients of all ages in contrast to the present study. After analyzing the selected 3796 children included in the current meta-analysis, we could conclude that MPV does not demonstrate any utility in AA diagnostics in children. More specifically, MPV did not improve diagnostic accuracy when comparing the values of 922 patients with AA and 874 healthy controls. Our results do not support the findings of the previous meta-analysis [33], as no statistically significant decreases in MPV levels could be detected in this cohort. The exact reason behind these contrasting results is not known. It is noteworthy that methodological differences among the included studies and inadequacies in sample processing may significantly hamper MPV measurements [34]. Both the meta-analyses (by Shen et al. and the current study) have highlighted substantial heterogeneity among the studies included in them. Therefore, well-designed prospective studies need to be conducted in the future.

Another finding in the current study was that MPV levels did not show any statistically significant variations among 1934 patients with acute appendicitis and 983 NSAP. This can be attributed to the non-specific decline in the levels of MPV in acute inflammatory conditions. Therefore, it would not be unreasonable to hypothesize that children with NSAP might have non-specific inflammatory conditions at the time of MPV measurement.

Antimicrobial therapy has been deemed safe and cost-effective in patients with confirmed uncomplicated appendicitis [35,36]. In children, antimicrobial therapy has also proven safe, but long-term outcomes regarding cost-effectiveness are still underway [37]. Currently, most children with AA are still treated by appendectomy, which entails some morbidity and costs. Early accurate diagnosis is essential to enable treatment options.

Previous research on the utility of biomarkers in diagnosing AA has failed to provide a definitive indicator for a complicated course. No single biomarker has thus far been identified, which would provide sufficient sensitivity and specificity for standalone use in the diagnosis of AA. Along these lines, while MPV remains to raise interest in inflammatory conditions, it fails to provide additional tools for diagnostic verification alongside clinical suspicion. The clinician treating pediatric patients has many diagnostic alternatives to use, and radiological findings frequently seem crucial. Combining a set of biomarkers with radiological investigations and clinical parameters enhances diagnostic accuracy, sensitivity, and specificity, but in younger patients, a consensus is still to be reached regarding optimal diagnosis. Applied mathematics has been suggested as a complementary tool for quick discrimination of complicated AA [38]. Given the current evidence regarding diagnostic accuracy, clinical vigilance remains the mainstay for proper treatment choices in pediatric AA. Clinical examination is limited by significant interobserver variability and low specificity [39].

Complicated appendicitis defines an entity for further research. While complicated appendicitis definitely entails perforated disease, it is not limited to that end. Interestingly, the current literature fails to provide much insight into usable biomarkers for identifying complicated diseases. Treatment alternatives for AA are a matter of intensive research and debate, but more focus should be directed on diagnostic strength to enable proper treatment decisions. Combining evidence from epidemiological, immunological, and molecular genetic studies indicates that AA is to be considered at histopathological levels as a phlegmonous vs. gangrenous disease [40,41,42,43]. The phlegmonous disease is thought to represent viral disease and gangrenous AA of bacterial etiology [42]. Although the immunologic reactions are stronger in gangrenous disease, at presentation, current biomarkers in clinical use seem unable to distinguish between the two disease presentations of AA. Artificial intelligence-based methods are under investigation for future discriminatory diagnostics, but they will most probably need to be combined with current modalities [43].

The results of this study must be interpreted within the context of a few limitations. First, most of the included studies had a retrospective study design. Second, a non-uniform reporting of the outcomes was observed among the included studies. Third, significantly high heterogeneity was observed amongst these studies while evaluating the outcomes. Fourth, being a non-specific marker of inflammation, MPV levels tend to rise in all inflammatory conditions. Finally, rather than focusing on a single biomarker, further studies must explore the combined sensitivity and specificity of a panel of biomarkers.

## 5. Conclusions

While MPV levels seem to have some utility in adults with AA, the present meta-analysis results indicate that MPV levels should not dictate treatment options in pediatric AA. There were no significant differences in MPV levels among children with AA versus healthy controls and children with AA versus non-AA. However, due to the substantial heterogeneity among the included studies, well-designed prospective studies need to be conducted before any definite conclusions are drawn.

## Figures and Tables

**Table 1:** Characteristics of the included studies.

Author, Year	Study Design	Sample Size		
		AA	Non-AA	Controls
Bilici et al., 2011 [13]	Retrospective	100	-	100
Uyanik et al., 2012 [7]	Retrospective	305	-	305
Nazik et al., 2017 [14]	Prospective	30	-	33
Yilmaz et al., 2017 [15]	Retrospective	628	30	-
Yazar et al., 2018 [16]	Cross-sectional	170	30	-
Nia et al., 2018 [17]	Case-control	60	60	-
Bozlu et al., 2019 [18]	Retrospective	141	46	100
Tuncer et al., 2019 [19]	Retrospective	137	65	-
Oktay et al., 2020 [20]	Retrospective	103	34	-
Sengul et al., 2020 [21]	Retrospective	205	30	-
Du et al., 2020 [22]	Prospective	92	-	90
Tartar et al., 2020 [23]	Retrospective	186	15	-
Duman et al., 2022 [24]	Retrospective	254	197	150
Dooki at al., 2022 [25]	Retrospective	50	50	-

**Table 2:** Methodological assessment for the included studies by Observer 1 and Observer 2.

**Study**	**Reporting**	**External Validity**	**Internal Validity-Bias**	**Internal Validity-Confounding**	**Power**	**Total Scores**
**Methodological assessment by Observer 1**						
Bilici, 2011 [13]	11	3	4	4	5	27
Uyanik, 2012 [7]	10	3	5	4	5	27
Nazik, 2017 [14]	9	3	4	3	5	24
Yilmaz, 2017 [15]	11	3	4	4	5	27
Yazar, 2018 [16]	9	3	5	3	5	25
Nia, 2018 [17]	11	3	5	4	5	28
Bozlu, 2019 [18]	9	3	4	3	5	24
Tuncer, 2019 [19]	11	3	5	4	5	28
Oktay, 2020 [20]	11	3	5	4	5	28
Sengul, 2020 [21]	10	3	5	4	5	27
Du, 2020 [22]	9	3	5	3	5	25
Tartar, 2020 [23]	11	3	5	4	2	25
Duman, 2022 [24]	9	3	5	3	5	25
Dooki, 2022 [25]	9	3	4	3	5	24
**Methodological assessment by Observer 2**						
Bilici, 2011 [13]	10	3	5	3	5	26
Uyanik, 2012 [7]	9	3	5	3	5	25
Nazik, 2017 [14]	9	3	5	3	5	25
Yilmaz, 2017 [15]	11	3	5	4	5	28
Yazar, 2018 [16]	9	3	5	3	5	25
Nia, 2018 [17]	10	3	5	4	5	27
Bozlu, 2019 [18]	9	3	5	3	5	25
Tuncer, 2019 [19]	11	3	5	4	5	28
Oktay, 2020 [20]	10	3	5	3	5	26
Sengul, 2020 [21]	9	3	5	3	5	25
Du, 2020 [22]	9	3	5	3	5	25
Tartar, 2020 [23]	10	3	5	3	2	23
Duman, 2022 [24]	9	3	5	3	5	25
Dooki, 2022 [25]	9	3	5	3	5	25
**Inter-Observer Agreement**						
**Study**	**Observer 1**	**Observer 2**	**Mean**	**Kappa Value**	***p*-value**	
Bilici, 2011 [13]	27	26	26.5	0.928	<0.0001	
Uyanik, 2012 [17]	27	25	26			
Nazik, 2017 [14]	24	25	24.5			
Yilmaz, 2017 [15]	27	28	27.5			
Yazar, 2018 [16]	25	25	25			
Nia, 2018 [17]	28	27	27.5			
Bozlu, 2019 [18]	24	25	24.5			
Tuncer, 2019 [19]	28	28	28			
Oktay, 2020 [20]	28	26	27			
Sengul, 2020 [21]	27	25	26			
Du, 2020 [22]	25	25	25			
Tartar, 2020 [23]	25	23	24			
Duman, 2022 [24]	25	25	25			
Dooki, 2022 [25]	24	25	24.5			

## Data Availability

The datasets used and/or analyzed during the current study are available from the corresponding author on reasonable request.

## References

[1] Lounis Y., Hugo J., Demarche M., Seghaye M.-C. (2020). Influence of age on clinical presentation, diagnosis delay and outcome in preschool children with acute appendicitis. BMC Pediatr..

[2] Nance M.L., Adamson W.T., Hedrick H. (2000). Appendicitis in the young child: A continuing diagnostic challenge. Pediatr. Emerg. Care.

[3] Lindestam U., Almström M., Jacks J., Malmquist P., Lönnqvist P.-A., Lagerbon Jensen B., Carlström M., Krmar R.T., Svensson J.F., Norberg Å. (2020). Low plasma sodium concentration predicts perforated acute appendicitis in children: A prospective diagnostic accuracy study. Eur. J. Pediatr. Surg.

[4] Yang J., Liu C., He Y., Cai Z. (2019). Laboratory markers in the prediction of acute perforated appendicitis in children. Emerg. Med. Int..

[5] Biricik S., Narci H., Dundar G.A., Ayrik C., Türkmenoglu M.Ö. (2019). Mean platelet volume and the ratio of mean platelet volume count in the diagnosis of acute appendicitis. Am. J. Emerg. Med..

[6] Anand S., Krishnan N., Jukic M., Krizanac Z., Llorente Munoz C.M., Pogorelic Z. (2022). Utility of red cell distribution width (RDW) as a noninvasive biomarker for the diagnosis of acute appendicitis: A systematic review and meta-analysis of 5222 cases. Diagnostics.

[7] Uyanik B., Kavalci C., Arslan D.E., Yilmaz F., Aslan O., Dede S., Bakir F. (2012). Role of mean platelet volume in diagnosis of childhood acute appendicitis. Emerg. Med. Int..

[8] Fan Z., Zhang Y., Pan J., Wang S. (2017). Acute appendicitis and mean platelet volume: A systematic review and meta-analysis. Ann. Clin. Lab. Sci..

[9] Moher D., Liberati A., Tetzlaff J., Altman D.G. (2009). Preferred reporting items for systematic reviews and meta-analyses: The PRISMA statement. BMJ.

[10] Downs S.H., Black N. (1998). The feasibility of creating a checklist for the assessment of the methodological quality both of randomised and non-randomised studies of health care interventions. J. Epidemiol. Community Health.

[11] Landis J.R., Koch G.G. (1977). The measurement of observer agreement for categorical data. Biometrics.

[12] (2021). Cochrane Handbook for Systematic Reviews of Interventions (Version 6.2) [Internet]. Cochrane. www.training.cochrane.org/handbook.

[13] Bilici S., Sekmenli T., Göksu M., Melek M., Avci V. (2011). Mean platelet volume in diagnosis of acute appendicitis in children. Afr. Health Sci..

[14] Nazik S., Avci V., Küskü Kiraz Z. (2017). Ischemia-modified albumin and other inflammatory markers in the diagnosis of appendicitis in children. Ulus. Travma Acil Cerrahi Derg..

[15] Yilmaz B.K., Ayhan Acar Y. (2017). Investigation of the Diagnostic Value of Neutrophil to Lymphocyte Ratio in Pediatric Appendicitis Cases. Iran. J. Pediatr..

[16] Yazar A.S., Erdogan S., Sahin C., Güven S. (2018). Reliability of ultrasonography and the Alvarado scoring system in acute appendicitis. Turk. J. Pediatr..

[17] Nia A.A., Zareifar P. (2018). Mean platelet volume (MPV) in children with acute appendicitis. J. Pioneer Med. Sci..

[18] Bozlu G., Akar A., Durak F., Kuyucu N. (2019). Role of mean platelet volume-to-lymphocyte ration in the diagnosis of childhood appendicitis. Arch. Argent Pediatr..

[19] Tuncer A.A., Cavus S., Balcioglu A., Silay S., Demiralp I., Calkan E., Altin M.A., Eryilmaz E., Karaisaoglu A.O., Bukulmez A. (2019). Can mean platelet volume, neutrophil-to-lymphocyte, lymphocyte-to-monocyte, platelet-to-lymphocyte ratios be favourable predictors for the differenbtial diagnosis of appendicitis?. J. Pak. Med. Assoc..

[20] Oktay M.M., Bogan M., Colak S.T., Sabak M. (2020). Evaluation of the diagnostic value of platelet indices in pediatric acute appendicitis. J. Int. Med. Res..

[21] Sengul S., Guler Y., Calis H., Karabulut Z. (2020). The Role of Serum Laboratory Biomarkers for Complicated and Uncomplicated Appendicitis in Adolescents. J. Coll. Physicians Surg. Pak..

[22] Du X., Chen Y., Zhu J., Bai Z., Hua J., Li Y., Lv H., Zhang G. (2020). sB7H3 in Children with Acute Appendicitis: Its Diagnostic Value and Association with Histological Findings. J. Immunol. Res..

[23] Tartar T., Bakal U., Sarac M., Aydin S., Kazez A. (2020). Diagnostic value of laboratory results in children with acute appendicitis. Turk. J. Biochem..

[24] Duman L., Karaibrahimoglu A., Büyükyavuz B.I., Savas M.C. (2022). Diagnostic Value of Monocyte-to-Lymphocyte Ratio Against Other Biomarkers in Children with Appendicitis. Pediatr. Emerg. Care.

[25] Dooki M.E., Nezhadan M., Mehrabani S., Osia S., Hadipoor A., Hajiahmadi M., Mohammadi M. (2022). Diagnostic accuracy of laboratory markers for diagnosis of acute appendicitis in children. Wien. Med. Wochenschr..

[26] Salminen P., Sippola S., Haijanen J., Nordström P., Rantanen T., Rautio T., Sallinen V., Löyttyniemi E., Hurme S., Tammilehto V. (2022). Antibiotics versus placebo in adults with CT-confirmed uncomplicated acute appendicitis (APPAC III): Randomized double-blind superiority trial. Br. J. Surg..

[27] Livingston E.H., Woodward W.A., Sarosi G.A., Haley R.W. (2007). Disconnect between incidence of nonperforated and perforated appendicitis: Implications for pathophysiology and management. Ann. Surg..

[28] Kim H.Y., Park J.H., Lee S.S., Jeon J.J., Yoon C.K., Lee K.H. (2021). Differentiation between complicated and uncomplicated appendicitis: Diagnostic model development and validation study. Abdom. Radiol..

[29] Nijssen D.J., Amstel P., Schuppen J., Eeftinck Shattenkerk L.D., Gorter R.R., Bakx R. (2021). Accuracy of ultrasonography for differentiating between simple and complex appendicitis in children. Pediatr. Surg. Int..

[30] D’Souza N., Hicks G., Beable R., Higginson A., Rud B. (2021). Magnetic resonance imaging (MRI) for diagnosis of acute appendicitis. Cochrane Database Syst. Rev..

[31] Erdem H., Aktimur R., Cetinkunar S., Reyhan E., Gokler C., Irkorucu O., Sozen S. (2015). Evaluation of mean platelet volume as a diagnostic biomarker in acute appendicitis. Int. J. Clin. Exp. Med..

[32] Tullavardhana T., Sanguanlosit S., Chartkitchareon A. (2021). Role of platelet indices as a biomarker for the diagnosis of acute appendicitis and as a predictor of complicated appendicitis: A meta-analysis. Ann. Med. Surg. (Lond.).

[33] Shen G., Li S., Shao Z., Liu L., Liu Q., Yu H., Wang H., Mei Z. (2021). Platelet indices in patients with acute appendicitis: A systematic review with meta-analysis. Updates Surg..

[34] Beyan E., Beyan C. (2022). Mean platelet volume may not decrease in patients with acute appendicitis. Updates Surg..

[35] Salminen P., Tuominen R., Paajanen H., Rautio T., Nordström P., Aarnio M., Rantanen T., Hurme S., Mecklin J.-P., Sand J. (2018). Five-year-follow-up of antibiotic therapy for uncomplicated acute appendicitis in the APPAC randomized clinical trial. JAMA.

[36] Haijanen J., Sippola S., Tuominen R., Grönroos J., Paajanen H., Rautio T., Nordström P., Aarnio M., Rantanen T., Hurme S. (2019). Cost analysis of antibiotic therapy versus appendectomy for treatment of uncomplicated acute appendicitis: 5-year results of the APPAC randomized clinical trial. PLoS ONE.

[37] Minneci P.C., Hade E.M., Lawrence A.E., Sebastiao Y.V., Saito J.M., Mak G.Z., Fox C., Hirschl R.B., Gadepalli S., Helmrath M.A. (2020). Association of nonoperative management using antibiotic therapy vs laparoscopic appendectomy with treatment success and disability days in children with uncomplicated appendicitis. JAMA.

[38] Zachos K., Fouzas S., Kolonitsiou F., Skiadopoulos S., Gkentzi D., Karatza A., Marangos M., Dimitriou G., Georgiou G., Sinopidis X. (2021). Prediction of complicated appendicitis risk in children. Eur. Rev. Med. Pharmacol. Sci..

[39] Banabbas R., Hanna M., Shah J., Sinert R. (2017). Diagnostic accuracy of history, physical examination, laboratory tests, and point-of-care ultrasound for pediatric acute appendicitis in the emergency department: A systematic review and meta-analysis. Acad. Emerg. Med..

[40] Rubér M., Berg A., Ekerfelt C., Olaison G., Andersson R.E. (2006). Different cytokine profiles in patients with a history of gangrenous or phlegmonous appendicitis. Clin. Exp. Immunol..

[41] Rubér M., Andersson M., Petersson B.F., Olaison G., Andersson R.E., Ekerfeldt C. (2010). Systemic Th17-like cytokine pattern in gangrenous appendicitis but not in phlegmonous appendicitis. Surgery.

[42] Kiss N., Minderjahn M.I., Reismann J., Svensson J., Wester T., Hauptmann K., Schad M., Kallarackal J., von Bernuth H., Reismann M. (2021). The use of gene-expression profiling to idenfify candidate genes for pretherapeutic patient classification in acute appendicitis. BJS Open.

[43] Reismann J., Kiss N., Reismann M. (2021). The application of artificial intelligence methods to gene expression data for differentiation of uncomplicated and complicated appendicitis in children and adolescents—A proof of concept study. BMC Pediatr..

